# The Inhibitory Effect and Mechanism of the Histidine-Rich Peptide rAj-HRP from *Apostichopus japonicus* on Human Colon Cancer HCT116 Cells

**DOI:** 10.3390/molecules29215214

**Published:** 2024-11-04

**Authors:** Yuebin Zhang, Shan Gao, Jiaming Mao, Yuyao Song, Xueting Wang, Jingwei Jiang, Li Lv, Zunchun Zhou, Jihong Wang

**Affiliations:** 1 School of Life Sciences, Liaoning Normal University, Dalian 116081, China; yuebinz@hotmail.com (Y.Z.); jiamingmao@hotmail.com (J.M.); yolanda_songyy@hotmail.com (Y.S.); 2 Liaoning Key Lab of Marine Fishery Molecular Biology, Liaoning Ocean and Fisheries Science Research Institute, Dalian 116023, China; gs_070920@hotmail.com (S.G.); weijingjiang@live.cn (J.J.); 3 Department of Pharmacology, College of Pharmacy, Dalian Medical University, Dalian 116044, China; wxt1344943824@hotmail.com (X.W.); lvli@dmu.edu.cn (L.L.)

**Keywords:** rAj-HRP, *Apostichopus japonicus*, histidine-rich peptide, HCT116 cells, EGFR

## Abstract

Colon cancer is a common and lethal malignancy, ranking second in global cancer-related mortality, highlighting the urgent need for novel targeted therapies. The sea cucumber (*Apostichopus japonicus*) is a marine organism known for its medicinal properties. After conducting a bioinformatics analysis of the cDNA library of *Apostichopus japonicus*, we found and cloned a cDNA sequence encoding histidine-rich peptides, and the recombinant peptide was named rAj-HRP. Human histidine-rich peptides are known for their anti-cancer properties, raising questions as to whether rAj-HRP might exhibit similar effects. To investigate whether rAj-HRP can inhibit colon cancer, we used human colon cancer HCT116 cells as a model and studied the tumor suppressive activity in vitro and in vivo. The results showed that rAj-HRP inhibited HCT116 cell proliferation, migration, and adhesion to extracellular matrix (ECM) proteins in vitro. It also disrupted the cytoskeleton and induced apoptosis in these cells. In vivo, rAj-HRP significantly inhibited the growth of HCT116 tumors in BALB/c mice, reducing tumor volume and weight without affecting the body weight of the tumor-bearing mice. Western blot analysis showed that rAj-HRP inhibited HCT116 cell proliferation and induced apoptosis by upregulating BAX and promoting PARP zymogen degradation. Additionally, rAj-HRP inhibited HCT116 cell adhesion and migration by reducing MMP2 levels. Further research showed that rAj-HRP downregulated EGFR expression in HCT116 cells and inhibited key downstream molecules, including AKT, P-AKT, PLCγ, P38 MAPK, and c-Jun. In conclusion, rAj-HRP exhibits significant inhibitory effects on HCT116 cells in both in vitro and in vivo, primarily through the EGFR and apoptosis pathways. These findings suggest that rAj-HRP has the potential as a novel targeted therapy for colon cancer.

## 1. Introduction

Colon cancer (CRC) is the third most common cancer in the world, causing more than 5 million deaths annually. Notably, colon cancer was the second leading cause of cancer deaths in 2020 [1]. Although 25–50% of patients are screened in the early stages of cancer, its recurrence or metastasis results in a low patient survival rate within 5 years, and there is a significant trend towards younger age of onset [2,3]. It is predicted that people under 50 years of age will account for one in ten cases of colon cancer and one in four cases of rectal cancer [4]. Currently, the mainstay of medical interventions for cancer encompasses a wide range of surgical interventions, chemotherapy, radiation therapy, and targeted therapies [5,6]. Surgical resection is still the dominant treatment strategy used at the initial stage of diagnosis. However, even though surgery is effective in removing the tumor and its metastatic foci and significantly improves the survival of colorectal cancer (CRC) patients, approximately one quarter of patients are in advanced stages of the disease at the time of diagnosis. The characteristics of advanced tumors, including aggressive spread, distant metastasis, poor prognosis, and resistance to treatment, make surgical intervention ineffective. This results in approximately half of the cases being diagnosed with CRC, with interventions considered ineffective, resulting in life-threatening outcomes for approximately half of the patients [6,7,8]. Therefore, more effective treatments and safer drugs are urgently needed to improve the clinical prognosis of CRC patients.

The epidermal growth factor receptor (EGFR) consists of extracellular, transmembrane, and intracellular structural domains that regulate cell proliferation, migration, survival, and differentiation. Aberrant EGFR signaling is associated with poor prognosis and leads to the development of a variety of tumors, including lung and colorectal cancer (CRC) [9,10]. With the success of the anti-EGFR drug cetuximab and the anti-angiogenic drug bevacizumab, new drugs that block different key pathways and immune checkpoints are emerging at an unprecedented rate [11]. Studies have shown that the intake of foods with anti-cancer properties can significantly reduce the incidence of cancer. Moreover, natural products and their derivative extracts show great potential in reducing cancer risk. Due to their minimal damage to normal tissues, they are the first line of hope for treatments with minimal side effects [12].

A significant portion of Earth’s organisms live in the ocean, where the unique environment produces a range of bioactive substances, drawing growing scientific interest in marine resources [13,14]. *Apostichopus japonicus*, recognized for its rich nutritional content and high medicinal value, attracted our interest. We identified a sequence in its cDNA library that encodes a histidine-rich peptide. After cloning, expression, and purification, we successfully obtained a purified recombinant peptide named rAj-HRP. Bioinformatics analysis reveals that histidine-rich peptides from human tissues have activities including angiogenesis inhibition, tumor cell proliferation suppression, and antibacterial effects [15,16,17,18,19]. Thus, we explored whether rAj-HRP might also exhibit anti-tumor properties. Due to the urgent need for targeted therapies in colon cancer, we used human colon cancer HCT116 cells as a model to evaluate the functions of rAj-HRP both in vivo and in vitro and to investigate its mechanism of action.

## 2. Results

### 2.1. Generation of rAj-HRP Peptide

**Figure 1:**
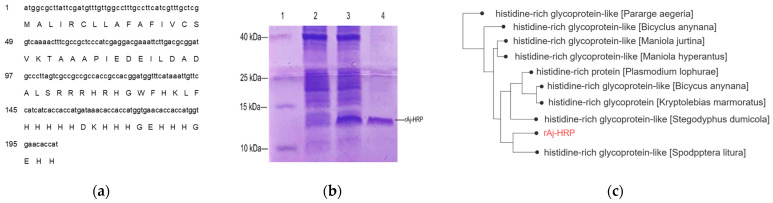
Sequence, purification, and BLAST results of rAj-HRP. (**a**) The cDNA sequence and deduced amino acid sequence of rAj-HRP; (**b**) Tricine-SDS-PAGE analysis showing the purification of recombinant rAj-HRP. Lane 1: marker; Lane 2: uninduced expression of recombinant B21; Lane 3: induced expression of recombinant B21; Lane 4: purified rAj-HRP; (**c**) phylogenetic tree analysis of histidine-rich glycoprotein-like from rAj-HRP BLAST results. The text marked in red represents the histidine-rich peptide rAj-HRP from *Apostichopus japonicus*. The amino acid sequence of rAj-HRP shares around 60% similarity with histidine-rich glycoprotein-like from various species such as *Pararge aegeria*, *Bicyclus anynana*, *Maniola jurtina*, *Maniola hyperantus*, *Plasmodium lophurae*, *Kryptolebias marmoratus*, *Stegodyphus dumicola*, and *Spodoptera litura*.

The cDNA of rAj-HRP from the cDNA library of *Apostichopus japonicus* was 201 bp long, and the deduced amino acid sequence contained 67 amino acids, including 16 histidines (Figure 1a). Due to the high histidine content in the primary sequence of rAj-HRP, we expected to use its own histidine sequence for nickel ion affinity chromatography during purification. Therefore, when the cDNA of rAj-HRP was constructed into the pET23b vector, we added a stop codon at the end of the cDNA to obtain the recombinant peptide without the C-terminal His-tag. The results showed that recombinant rAj-HRP was expressed as a soluble protein, and the purified production was obtained by using histidine affinity chromatography with a nickel column. Purification was confirmed using Tricine-SDS-PAGE, which showed that rAj-HRP reached a purity of up to 95% (Figure 1b). Bioinformatics analysis revealed that the molecular weight of rAj-HRP was 7.99 kDa, and the isoelectric point was 7.31. The rAj-HRP sequence shares around 60% similarity with histidine-rich glycoprotein-like (HRG-like) proteins from species such as *Pararge aegeria*, *Plasmodium lophurae*, and *Spodoptera litura* (Figure 1c).

#### 2.1.1. rAj-HRP Effectively Inhibits Cell Proliferation

**Figure 2:**
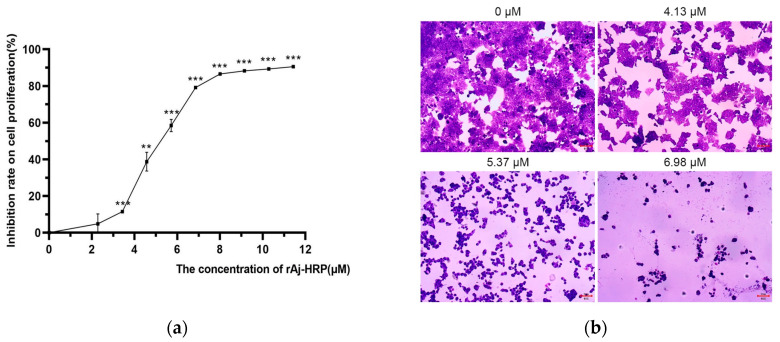
Inhibitory effect of rAj-HRP on the proliferation and morphology of HCT116 cells. (**a**) CCK-8 assay results (*n* = 3) showing the inhibitory effect of rAj-HRP on HCT116 cell proliferation. Significant differences between the rAj-HRP-treated group and the control group (treated with empty medium) are indicated by *, ** *p* < 0.01, and *** *p* < 0.001. (**b**) Wright–Giemsa staining illustrating the effect of rAj-HRP on HCT116 cell morphology (Olympus digital imaging microscope, Tokyo, Janpan); 200× magnification; the bar indicates 2 μm.

To investigate the effect of rAj-HRP on colon cancer cell proliferation, the CCK-8 assay was employed to measure cell survival 24 h after treatment with varying concentrations of rAj-HRP. The CCK-8 assay results demonstrated that rAj-HRP significantly inhibited HCT116 cell proliferation, with the effect intensifying in a dose-dependent manner as rAj-HRP concentration increased. The half-maximal inhibitory concentration (IC_50_) of rAj-HRP for HCT116 cells was calculated as 5.21 μM using GraphPad Prism 8.0 (Figure 2a).

The effect of rAj-HRP on HCT116 cell morphology was assessed via Wright–Giemsa staining. As shown in Figure 2b, in cells untreated or treated with rAj-HRP for 24 h, the control group of the HCT116 cells exhibited high density, growing in clusters and maintaining good condition. With increasing concentrations of rAj-HRP, HCT116 cell density gradually decreased, intercellular connections weakened, and cells transitioned from clusters to more dispersed single cells. The cells also exhibited morphological changes, including edge shrinkage and nuclear condensation, indicating that rAj-HRP induced the apoptosis of HCT116 in a dose-dependent manner.

#### 2.1.2. rAj-HRP Inhibits Cell Migration

**Figure 3:**
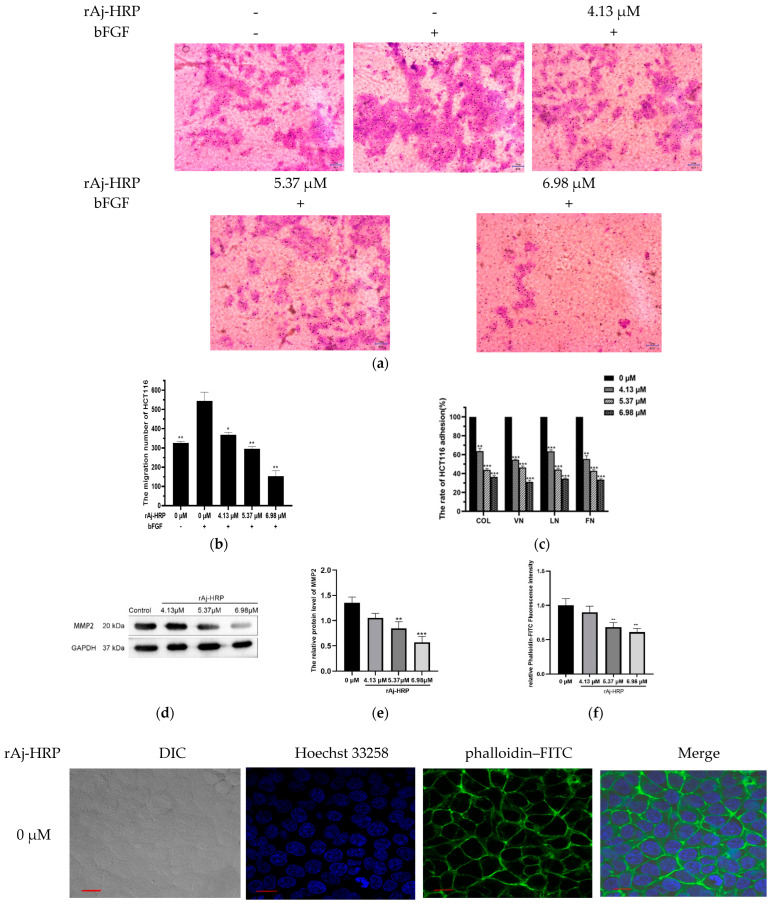

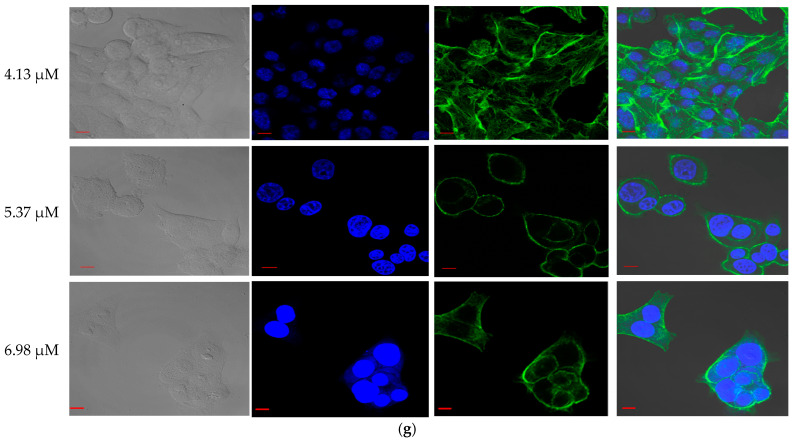
Inhibition of HCT116 cell migration by rAj-HRP via ECM adhesion, cytoskeleton disruption, and MMP2 downregulation. (**a**) Effect of rAj-HRP on HCT116 cell migration towards bFGF: HCT116 cell migration was evaluated using the Transwell assay, with each experiment conducted in triplicate. (**b**) Significant differences in migration between groups treated with bFGF or rAj-HRP and the bFGF+ control group are marked by * (* *p* < 0.05, and ** *p* < 0.01; the bar indicates 2 μm. (**c**) The effect of rAj-HRP on the adhesion to ECM proteins: HCT116 cell adhesion to ECM proteins in the presence of rAj-HRP was quantified using the CCK-8 assay. Adhesion rates were calculated using the formula provided in the Materials and Methods Section. Each experiment was performed in triplicate. Significant differences in adhesion rates between the blank medium or rAj-HRP-treated groups are denoted by * (** *p* < 0.01, and *** *p* < 0.001). (**d**) Western blot results; the downregulation of MMP2 expression by rAj-HRP: Western blot analysis was conducted to determine MMP2 protein levels in HCT116 cells treated with either blank medium or rAj-HRP (4.13 μM, 5.37 μM, or 6.98 μM), with GAPDH as a control. (**e**) Statistical chart displaying the relative gray value of MMP2 (MMP2/GAPDH) (*n* = 3). Significant differences between the rAj-HRP-treated and control groups are represented by * (** *p* < 0.01, and *** *p* < 0.001). (**f**) Cells were or were not treated with rAj-HRP, and the cytoskeleton was stained using phalloidin–FITC. Significant differences between the rAj-HRP-treated groups and control groups are represented by * (** *p* < 0.01). (**g**) Disruption of the HCT116 cell cytoskeleton by rAj-HRP: HCT116 cell morphology was observed using differential interference contrast (DIC) microscopy (first column). The blue signal indicates nuclei stained with Hoechst 33258 (second column), while the green signal indicates F-actin stained with FITC–phalloidin (third column). The fourth column displays merged images of the second and third columns. Images were captured using a Zeiss laser scanning confocal microscope (ZEISS, Oberkochen, German) at 630× magnification. The bar indicates 10 μm.

The ability to inhibit tumor cell migration is a critical measure of anti-cancer drug effectiveness. To assess whether rAj-HRP suppresses HCT116 cell migration, a Transwell assay was conducted. As shown in Figure 3a,b, in the control groups without drug treatment, the number of migrating cells was significantly lower in the group without bFGF in the lower chamber compared to the group with bFGF, indicating that bFGF significantly promotes HCT116 cell migration. In the treatment groups, the migration inhibition rates compared to the control with bFGF in the lower chamber were 32.35% at 4.13 μM rAj-HRP, 45.59% at 5.37 μM rAj-HRP, and 71.69% at 6.98 μM rAj-HRP. This indicates that rAj-HRP suppresses HCT116 cell migration in a dose-dependent manner in the presence of bFGF.

Considering rAj-HRP’s ability to inhibit HCT116 cell migration, we further explored the underlying mechanism. Since cell growth and migration are closely linked to extracellular matrix (ECM) proteins, we treated HCT116 cells with four classic adhesion molecules—collagen (COL), vitronectin (VN), laminin (LN), and fibronectin (FN)—using a gradient concentration of rAj-HRP (0 μM, 4.13 μM, 5.37 μM, or 6.98 μM) for 24 h to assess its impact on their adhesion capacity. The results showed that as the concentration of rAj-HRP increased, the adhesion rate of HCT116 cells to the four adhesion molecules progressively decreased, indicating that rAj-HRP inhibits HCT116 cell adhesion in a dose-dependent manner (Figure 3c).

The cytoskeleton is vital for maintaining cell shape and is involved in various types of cell movement, including mitosis and migration in tumor cells. The effect of rAj-HRP on the cytoskeleton of HCT116 cells was assessed via FITC–phalloidin staining. As shown in Figure 3f,g, after 24 h of rAj-HRP treatment, the control group exhibited clear cell contours and an intact cytoskeleton. With increasing concentrations of rAj-HRP, the microfilaments gradually disassembled, indicating that rAj-HRP disrupts the cytoskeleton of HCT116 cells in a dose-dependent manner, leading to apoptosis and inhibiting cell proliferation, adhesion, and migration.

Matrix metalloproteinase 2 (MMP2), a member of the MMP family, can cleave ECM components. MMP2 expression is closely associated with tumor cell migration and prognosis [20,21,22,23]. The effect of rAj-HRP on MMP2 expression in HCT116 cells was evaluated. As shown in Figure 3d,e, MMP2’s relative expression decreased with increasing concentrations of rAj-HRP, indicating that rAj-HRP downregulates MMP2 expression, thereby inhibiting HCT116 cell migration.

These findings suggest that rAj-HRP inhibits HCT116 cell migration by reducing cell adhesion to the ECM, disrupting the cytoskeleton, and downregulating MMP2 expression.

#### 2.1.3. rAj-HRP Induces Apoptosis

**Figure 4:**
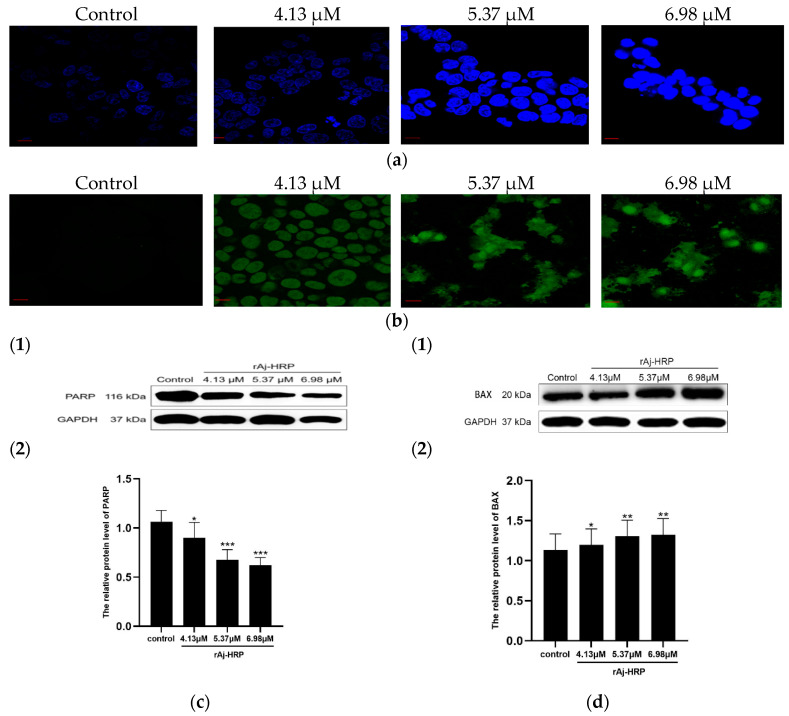
rAj-HRP induces apoptosis in HCT116 cells by modulating PARP and BAX expression. (**a**) The effect of rAj-HRP on HCT116 cell apoptosis assessed by the TUNEL assay (Zeiss laser scanning confocal microscope, 630× magnification; the bar indicates 10 μm). (**b**) The effect of rAj-HRP on HCT116 cell apoptosis assessed by Hoechst staining (Zeiss laser scanning confocal microscope, 630× magnification; the bar indicates 10 μm). (**c**) The effect of rAj-HRP on PARP expression in HCT116 cells: (1) Western blot results; (2) statistical chart showing the relative gray value of PARP (PARP/GAPDH) (*n* = 3; significance between rAj-HRP-treated groups and control groups is indicated by *, * *p* < 0.05, and *** *p* < 0.001). (**d**) The effect of rAj-HRP on BAX expression in HCT116 cells: (**1**) Western blot results; (**2**) statistical chart showing the relative gray value of BAX (BAX/GAPDH) (*n* = 3; significance between rAj-HRP-treated groups and control groups is indicated by *, * *p* < 0.05, and ** *p* < 0.01).

The induction of apoptosis and necrosis is a key mechanism by which drugs inhibit tumors and serves as a crucial indicator of drug efficacy [24,25]. In this study, we employed Hoechst 33258 staining and TdT-UTP terminal deoxynucleotidyl transferase dUTP nick end labeling (TUNEL) assays to assess the effects of rAj-HRP on HCT116 cells (Figure 4a). Hoechst 33258 staining revealed that the nuclei of rAj-HRP-treated cells showed stronger fluorescence compared to those treated with PBS, indicating chromatin condensation induced by rAj-HRP. TUNEL staining revealed an increased proportion of green FITC-labeled HCT116 cells in the rAj-HRP-treated group, suggesting that rAj-HRP induces DNA fragmentation (Figure 4b).

Poly (ADP-ribose) polymerase (PARP) is a nuclear enzyme essential for various cellular functions, especially in response to DNA damage [26]. PARP inhibitors (PARPis) are effective anti-cancer agents, with clinical trials demonstrating their ability to identify and repair DNA damage while maintaining good tolerability [27,28]. PARPis also show the potential for synergistic effects when combined with inhibitors of the PI3K/AKT pathway [29,30]. Our results indicate that rAj-HRP reduces the relative expression of PARP in a concentration-dependent manner (Figure 4c), suggesting that rAj-HRP downregulates PARP expression.

BAX, a pro-apoptotic protein in the Bcl-2 family, forms dimers with other family members to alter mitochondrial permeability, release cytochrome C, and activate the mitochondrial apoptosis pathway [31]. We evaluated the effect of rAj-HRP on BAX expression in HCT116 cells. The results in Figure 4d show that BAX expression increases with higher rAj-HRP concentrations. This suggests that rAj-HRP upregulates BAX, potentially inducing apoptosis in HCT116 cells through the mitochondrial pathway.

### 2.2. rAj-HRP Inhibited Tumor Growth In Vivo

**Figure 5:**
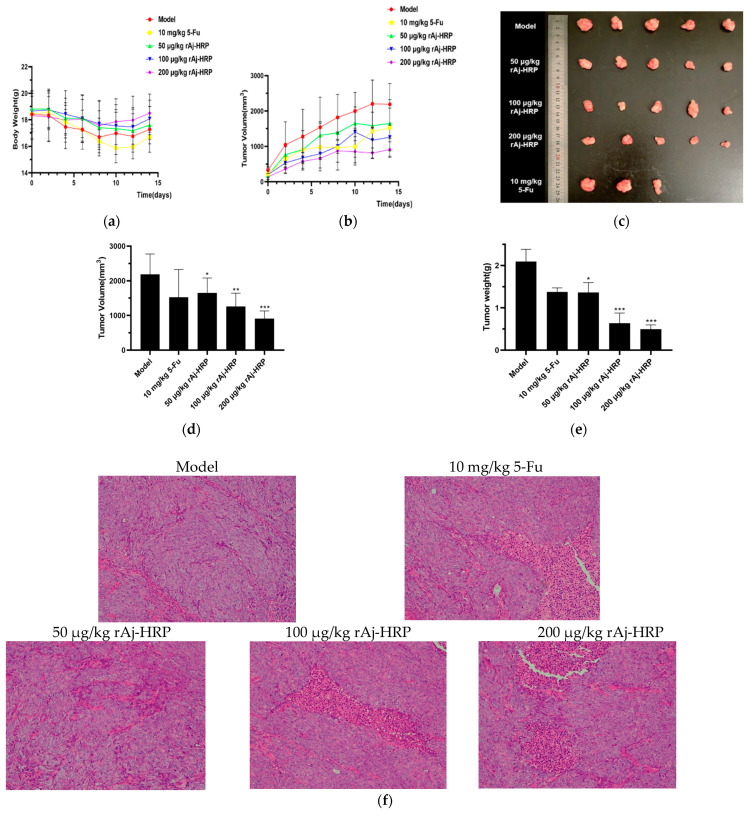
The effect of rAj-HRP on tumor growth in HCT116 xenograft BALB/c mice. (**a**) The effect of rAj-HRP on body weight: changes in body weight of HCT116 xenograft mice treated with rAj-HRP or 5-Fu (*n* = 5). (**b**) The effect of rAj-HRP on tumor volume growth: tumor volume growth trends in HCT116 xenograft mice treated with rAj-HRP or 5-Fu (*n* = 5). (**c**) Tumor images: representative images of tumors from different treatment groups. Unfortunately, two of the five nude mice injected with 5-Fu died 10 days after being administered the drug. (**d**) Tumor volume effect: tumor volumes in mice treated with rAj-HRP or 5-Fu compared to the control group (*n* = 5). Significant differences are indicated by *, * *p* < 0.05; ** *p* < 0.01, and *** *p* < 0.001. (**e**) The effect on tumor weight: tumor weights in mice treated with rAj-HRP or 5-Fu compared to the control group (*n* = 5). Significant differences are indicated by *, * *p* < 0.05 and *** *p* < 0.001. (**f**) HE staining of tumor sections: hematoxylin and eosin (HE) staining of HCT116 xenograft tumor sections from mice treated with rAj-HRP, observed using a Nikon inverted fluorescence microscope (Nikon, Tokyo, Iapan) at 200× magnification; the bar indicates 2 μm.

The above experiments confirm that rAj-HRP inhibits HCT116 cell proliferation in vitro. To assess its anti-tumor activity in vivo, we tested rAj-HRP using a nude mouse xenograft model. This study employed 5-Fluorouracil (5-Fu), a common anti-metabolite used in cancer chemotherapy, as the positive control in this study. Figure 5a shows that after 14 days of treatment, mice receiving rAj-HRP did not exhibit a significant reduction in body weight compared to those receiving normal saline. In contrast, mice treated with 5-Fu exhibited a marked decrease in body weight, indicating that rAj-HRP has lower toxicity than 5-Fu. Both rAj-HRP and 5-Fu treatments significantly reduced tumor growth in the HCT116 xenograft model. Tumor volume inhibition was notably greater in mice treated with 100 μg/kg or 200 μg/kg rAj-HRP compared to those treated with 10 mg/kg 5-Fu. The efficacy of 5-Fu was comparable to that of the lower dose of 50 μg/kg rAj-HRP (Figure 5b). After 14 days of treatment, the tumors were excised and weighed. The results indicate that rAj-HRP significantly reduced tumor weight and volume in a dose-dependent manner (Figure 5c–e, Table 1 and Table 2). Additionally, H&E staining of the tumor tissue sections revealed that tumors from the positive control and rAj-HRP-treated groups exhibited more disorganized structures and larger intercellular spaces compared to those from saline-treated mice. The degree of tumor disorganization and intercellular spacing increased with higher rAj-HRP concentrations.

### 2.3. Inhibition of EGFR Signaling by rAj-HRP in HCT116 Cells

**Figure 6:**
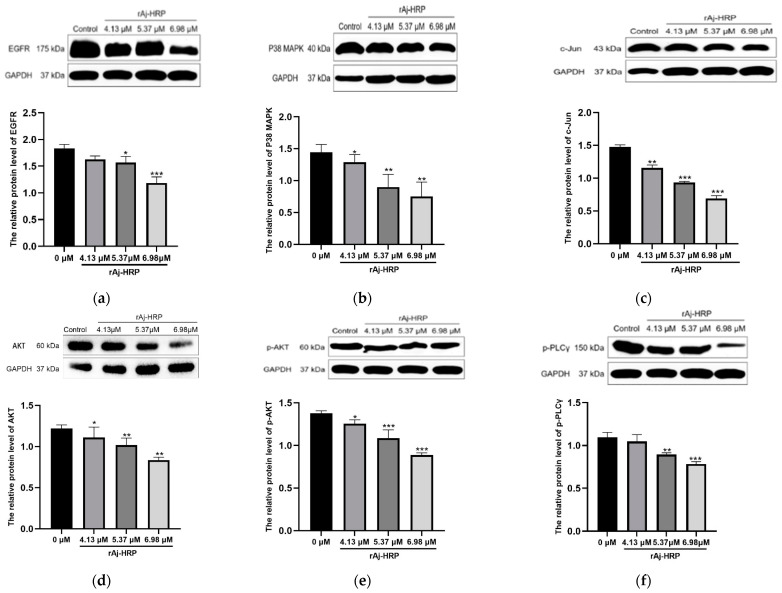
The effect of rAj-HRP on EGFR and its downstream signaling pathways in HCT116 cells. (**a**) EGFR expression: Western blot analysis of EGFR in HCT116 cells using GAPDH as a loading control. The histogram displays the relative gray value of EGFR (EGFR/GAPDH). (*n* = 3; significant differences between rAj-HRP-treated groups and the control group are indicated by *, * *p* < 0.05, and *** *p* < 0.001). (**b**) P38 MAPK expression: Western blot results for P38 MAPK. The histogram displays the relative gray values of P38 MAPK (P38 MAPK/GAPDH). (*n* = 3; significant differences between rAj-HRP-treated groups and the control group are indicated by *, * *p* < 0.05, ** *p* < 0.01, and *** *p* < 0.001). (**c**) c-Jun expression: Western blot results for c-Jun. The histogram displays the relative gray values of c-Jun (c-Jun/GAPDH). (*n* = 3; significant differences between rAj-HRP-treated groups and the control group are indicated by *, ** *p* < 0.01, and *** *p* < 0.001). (**d**) AKT expression: Western blot results for AKT. The histogram shows the relative gray values of AKT (AKT/GAPDH). (*n* = 3; significant differences between rAj-HRP-treated groups and the control group are indicated by *, * *p* < 0.05, and ** *p* < 0.01,). (**e**) p-AKT expression: Western blot results for AKT. The histogram shows the relative gray values of p-AKT (p-AKT/GAPDH). (*n* = 3; significant differences between rAj-HRP-treated groups and the control group are indicated by *, * *p* < 0.05, and *** *p* < 0.001). (**f**) p-PLCγ expression: Western blot results for p-PLCγ. The histogram displays the relative gray value of *p*-PLCγ (p-PLCγ/GAPDH). (*n* = 3; significant differences between rAj-HRP-treated groups and the control group are indicated by *, ** *p* < 0.01, and *** *p* < 0.001).

EGFR is a critical signaling molecule on the cell membrane that regulates essential cellular processes, including survival, proliferation, and differentiation [32,33,34]. An abnormal overexpression or mutation of EGFR in malignant tumors confers significant proliferative and angiogenic advantages to cancer cells [35]. Consequently, EGFR is regarded as a promising target for cancer therapy. In this study, we investigated the effect of rAj-HRP on EGFR expression in HCT116 cells by comparing cells treated with rAj-HRP to untreated controls. Our data reveal a significant decrease in EGFR expression with increasing rAj-HRP concentrations, indicating that rAj-HRP effectively downregulates EGFR (Figure 6a).

Upon activation by its ligands, EGFR transmits signals through several downstream pathways, including PI3K/AKT, Ras/MAPK, and PLC/PKC, mediating various physiological processes [35,36]. To explore the impact of rAj-HRP on these pathways, we treated HCT116 cells with gradient concentrations of rAj-HRP and measured key molecules involved in the following signaling pathways: AKT and its phosphorylated form (p-AKT) for the PI3K/AKT pathway; P38 MAPK and its associated transcription factor c-Jun for the Ras/MAPK pathway; and phosphorylated PLCγ (p-PLCγ) for the PLC/PKC pathway.

As shown in Figure 6, our results demonstrate that rAj-HRP significantly downregulates the expression of AKT, p-AKT, P38 MAPK, c-Jun, and p-PLCγ in a dose-dependent manner. This indicates that rAj-HRP inhibits EGFR signaling through the PI3K/AKT, Ras/MAPK, and PLC/PKC pathways. Therefore, the inhibitory effect of rAj-HRP on HCT116 cells is associated with the EGFR signaling pathway, making EGFR the primary target of rAj-HRP.

## 3. Discussion

rAj-HRP is a histidine-rich peptide derived from sea cucumbers (*Apostichopus japonicus*), consisting of 67 amino acids, 16 of which are histidine residues. Bioinformatics analysis shows that rAj-HRP shares over 60% sequence similarity with histidine-rich proteins from various species, including the butterfly *Pararge aegeria*, the malaria parasite *Plasmodium lophurae*, and the cotton leafworm *Spodoptera litura*. Although the functions of histidine-rich proteins from these species are not well documented, human histidine-rich glycoprotein (HRG) has been extensively studied.

Human HRG is a multi-domain plasma glycoprotein that interacts with various ligands to carry out diverse biological functions. Studies have demonstrated that HRG is involved in coagulation, fibrinolysis, cell adhesion, immune complex clearance, tumor angiogenesis regulation, and tumor immunity modulation [37,38,39]. HRG contains four main domains: an N-terminal region with two cystatin-like domains, a central histidine–proline-rich (His/Pro-rich) domain, and a C-terminal region. The His/Pro-rich domain, also known as the histidine-rich region (HRR), is essential for HRG’s function and promotes tumor-associated macrophage (TAM) polarization, thereby inhibiting tumor growth [40,41]. This led us to investigate whether rAj-HRP also exhibits anti-tumor properties.

Our study found that rAj-HRP significantly inhibits the proliferation, migration, and adhesion of HCT116 cells in a dose-dependent manner while also effectively reducing tumor weight and volume in vivo. Compared to the conventional chemotherapy drug 5-Fu, rAj-HRP minimally impacts the body weight of tumor-bearing mice, whereas 5-Fu causes significant weight loss due to its known side effects. This suggests that rAj-HRP is less toxic to mice. However, a thorough preclinical safety evaluation is necessary for a complete assessment. Furthermore, the anti-tumor efficacy of rAj-HRP at medium (100 μg/kg) and high (200 μg/kg) doses is significantly higher than that of 5-Fu (10 mg/kg), while the low dose (50 μg/kg) of rAj-HRP exhibits comparable efficacy to 5-Fu. This indicates that rAj-HRP may offer superior therapeutic potential for colorectal cancer compared to traditional treatments.

Given rAj-HRP’s potent inhibition of HCT116 cells, we also explored its mechanism of action. Inducing DNA damage is a well-established strategy for cancer treatment aimed at triggering apoptosis and inhibiting tumor cell proliferation [42]. We observed classic apoptotic features, such as chromatin condensation and DNA fragmentation, in rAj-HRP-treated HCT116 cells. Additionally, rAj-HRP induces apoptosis by downregulating PARP, a key factor in the death receptor pathway, and upregulating BAX, a pro-apoptotic protein in the mitochondrial pathway. Furthermore, rAj-HRP disrupts the arrangement of F-actin, reducing HCT116 cell migration and invasion. These findings suggest that rAj-HRP may be an effective apoptosis-inducing anti-cancer agent.

EGFR is a critical transmembrane receptor within the receptor tyrosine kinase (RTK) superfamily [43,44,45]. EGFR activation initiates intracellular signaling cascades, promoting cell proliferation, differentiation, migration, and survival [43,46]. Due to its association with tumor progression, EGFR has become a prominent target for anti-cancer therapies [43,45,47,48]. Studies have indicated that c-Met and EGFR are overexpressed in 78–80% of colon cancers, correlating with poor outcomes. Therefore, evaluating changes in key factors of EGFR downstream pathways, such as PI3K/AKT, Ras/MAPK, and PLC/PKC, is essential for understanding EGFR inhibitor efficacy [49,50,51]. Some EGFR inhibitors, such as cetuximab, block downstream signaling pathways by binding to EGFR [52,53,54,55,56]. Our results align with these findings, showing that rAj-HRP treatment reduces the phosphorylation levels of EGFR and its downstream molecules, including Akt and PLCγ. This suggests that rAj-HRP effectively inhibits EGFR signaling.

Since the PI3K/Akt and PLC/PKC pathways are crucial for regulating cell survival, proliferation, migration, and invasion [57,58], the anti-tumor activity of rAj-HRP may be attributed to its inhibition of EGFR and its downstream pathways. Additionally, the activation of P38 MAPK triggers a cascade of events, promoting tumor cell transcription through the transcription factor c-Jun [59,60,61]. Our study shows that rAj-HRP downregulates the expression of P38 MAPK and c-Jun in HCT116 cells in a dose-dependent manner. This indicates that rAj-HRP not only inhibits the Ras/MAPK pathway but also suppresses c-Jun, contributing to its overall anti-tumor effects.

Unfortunately, the specific mechanism by which rAj-HRP interacts with EGFR remains unexplored in this study. However, in 2019, our group reported that a histidine-rich peptide, rLj-112, inhibits tumor activity by binding to and internalizing EGFR on the cell surface [62]. Other studies suggest that EGFR and its ligands can be internalized via clathrin-coated pits and be either recycled to the membrane or degraded in lysosomes [63]. Given the high histidine content in rAj-HRP, it is plausible that it may similarly induce EGFR internalization in HCT116 cells, leading to tumor cell death. Further research is needed to confirm this mechanism.

## 4. Materials and Methods

### 4.1. Bioinformatics Analysis of rAj-HRP

Analysis was conducted using online tools available on the NCBI (https://www.ncbi.nlm.nih.gov), accessed on 13 April 2020.

### 4.2. Preparation of rAj-HRP

The rAj-HRP gene sequence was synthesized and cloned into the pET23b vector (pET23b-rAj-HRP, Genscript, Nanjing, China). The construct was transformed into Escherichia coli (*E. coli*) BL21 cells, and the recombinant peptide expression was induced with 1 mM IPTG at 30 °C for 15 h. The recombinant peptide was purified using a nickel affinity column (GE, Boston, MA, USA), and its purity was analyzed via Tricine-SDS-PAGE. The concentration of rAj-HRP was measured using the Coomassie Brilliant Blue (G250) assay.

### 4.3. Cell Culture

HCT116 cells were obtained from the Chinese Academy of Sciences (Shanghai, China). The cells were cultured in McCoy’s 5A medium (Meilunbio, Dalian, China) supplemented with 10% fetal bovine serum (FBS, life-iLab, Shanghai, China), 1:1 penicillin–streptomycin (life-iLab, Shanghai, China), and 1% L-glutamine (Meilunbio, Dalian, China) in a humidified incubator with 5% CO_2_ at 37 °C.

### 4.4. CCK-8 Assay

Cell viability was analyzed using the Cell Counting Kit-8 (Beyotime Biotechnology, Shanghai, China) according to the manufacturer’s protocols. HCT116 cells were seeded at 5 × 10^3^ cells/well in 96-well microplates (Corning, New York, NY, USA). Then, the cells were treated with varying concentrations of rAj-HRP (0 μM, 2.29 μM, 3.43 μM, 4.57 μM, 5.72 μM, 6.86 μM, 8.01 μM, 9.15 μM, or 10.29 μM) for 24 h. After treatment, the CCK-8 reagent was added to each well, and the cells were cultured for 2 h. Absorbance was measured at 450 nm with wells without cells as blanks. All the experiments were performed in triplicate.

### 4.5. Wright-Giemsa Staining Assay

The HCT116 cells were plated in 24-well plates containing slides and treated with varying concentrations of rAj-HRP (0 μM, 4.13 μM, 5.37 μM or 6.98 μM) for 24 h. Subsequently, the Wright–Giemsa Stain Kit (Nanjing Jiancheng Bioengineering Institute, Nanjing, China) was added to the 24-well plates and incubated at 37 °C for 5 min according to the manufacturer’s protocols. Then, the cells were observed using a microscope (OLYMPUS, Tokyo, Japan).

### 4.6. Adhesion Assay

To simulate in vivo conditions, four classic adhesion molecules (COL, VN, LN, and FN) were coated onto a 96-well plate at a concentration of 0.1 µg/µL. HCT116 cells were then added and treated with different concentrations of rAj-HRP (0 μM, 4.13 μM, 5.37 μM, or 6.98 μM) for 24 h. After 3 h, 10 μL of CCK-8 reagent was added to each well, and the absorbance was measured at 450 nm. Adhesion rates were calculated using the following formula:

$$Adhesive\;rate=\frac{{\bar{OD}}_{rAj-HRP\;treated}}{{\bar{OD}}_{Control\;Groups}}*100\%$$

where “—” means the average value of *OD*.

### 4.7. Migration Assay

Transwell migration assays were performed using inserts with 8.0 µm pore filters (Corning, USA). Untreated or rAj-HRP-treated cells were placed in the upper chamber with an empty medium, while the lower chamber contained FBS and bFGF at a final concentration of 3 ng/mL. After 20 h, the cells were fixed with 4% paraformaldehyde and stained with Wright–Giemsa dye. Then, the upper surface of the non-migrated membrane was wiped off. Finally, three fields were randomly selected and counted.

### 4.8. Hoechst 33258, TUNEL and Phalloidin–FITC Staining Assays

HCT116 cells were plated in 24-well plates containing slides and treated with different concentrations of rAj-HRP (0 μM, 4.13 μM, 5.37 μM, or 6.98 μM) for 24 h. Then, the cells were fixed with 4% paraformaldehyde for 30 min. Subsequently, the cells were stained using the Hoechst Staining Kit (Beyotime Biotechnology, Shanghai, China), Actin-Tracker Green (Beyotime Biotechnology, Shanghai, China), and the TUNEL Apoptosis Assay Kit (Beyotime Biotechnology, Shanghai, China) according to the manufacturer’s protocol. The assays were performed according to literature references.

### 4.9. Western Blot Analysis

The total protein from the treated cells was extracted using RIPA buffer (Solarbio, Beijing, China) containing PMSF (Solarbio, Beijing, China) at a ratio of 1:100. The sample was then centrifuged at 12,000 rpm for 10 min at 4 °C. The supernatant was collected, and the total protein content was measured using the BCA assay (Beyotime, Shanghai, China). Equal amounts of protein (20 μg/lane) were loaded onto SDS-PAGE gels and transferred to 0.45 μm PVDF membranes. The membranes were blocked with 5% skim milk (5% skim milk powder in TBST) and then incubated with primary antibodies overnight at 4 °C. The membrane was washed four times with TBST for 10 min each time and then incubated with the secondary antibody for 1 h at room temperature. After washing, the target bands were visualized using a chemiluminescence gel imaging system (Bio-Rad, Hercules, CA, USA).

The antibodies used include anti-GAPDH (Cell Signaling Technology, Boston, MA, USA, 1:4000), anti-EGF (Cell Signaling Technology, USA, 1:1000), anti-Akt (Cell Signaling Technology, USA, 1:1000), anti-phospho-Akt (Cell Signaling Technology, USA, 1:1000), anti-phospho-PLCγ1 (Cell Signaling Technology, USA, 1:1000), anti-PARP (Proteintech, Wuhan, China, 1:2000), anti-c-Jun (Proteintech, China, 1:2000), anti-p38 MAPK (Proteintech, China, 1:2000), anti-BAX (Proteintech, China, 1:2000), and anti-MMP2 (Proteintech, China, 1:1000). Horseradish peroxidase-conjugated goat anti-rabbit (Zsbio, Beijing, China, 1:5000) and horseradish peroxidase-conjugated goat anti-mouse (Zsbio, China, 1:5000) were also used.

### 4.10. Animal Tumor Model

Male BALB/c nude mice, aged 6–8 weeks, were purchased from Jiangsu Huachuang Sinoe Pharmaceutical Technology Co., Ltd. (Permit number: SCXK2020-0009, jJiangsu, China) and maintained at the School of Pharmacy, Dalian Medical University. All animal experimental protocols were approved by the Animal Care and Ethics Committee of Dalian Medical University. One week after subcutaneously injecting the mice with 1 × 10^7^ HCT116 cells in the right axillary region, the mice were divided into five groups (five mice per group). They received daily intraperitoneal injections of either normal saline, rAj-HRP (50 μg/kg, 100 μg/kg, or 200 μg/kg), or 5-Fu (10 mg/kg) for 14 days. Tumor size was measured daily using a caliper, recording the longest (L) and shortest (W) dimensions, and tumor volume (V) was calculated using the formula V (mm^3^) = 1/2 × L × W^2^. The care and use of the experimental animals followed established guidelines.

### 4.11. HE Assays

After the mice were sacrificed, the tumors were promptly excised and immersed in 10% formaldehyde for 24 h to fix the tissue. Following fixation, the tissue samples were dehydrated through a series of ethanol concentrations (80%, 90%, 95%, or 100%) and then embedded in paraffin. Sections that were 5 μm thick were cut and stained with hematoxylin and eosin (H&E) (Solarbio, Beijing, China). The stained sections were subsequently examined and imaged using an inverted microscope.

### 4.12. Statistical Analysis

The data were analyzed using GraphPad Prism 8.0. The results are presented as means ± SD from at least three independent experiments. Statistical significance was analyzed using one-way analysis of variance (ANOVA), with significance levels denoted as * *p* < 0.05, ** *p* < 0.01, and *** *p* < 0.001.

## 5. Conclusions

The histidine-rich peptide rAj-HRP, derived from *Apostichopus japonicus*, has demonstrated significant anti-cancer activity against HCT116 human colon cancer cells in both in vitro and in vivo models. Its anti-tumor effects are primarily attributed to the inhibition of the EGFR signaling pathway and the induction of apoptotic cell death. These findings underscore the therapeutic potential of rAj-HRP as a novel, targeted agent for treating colorectal cancer, suggesting promise for the development of more effective colorectal cancer treatments.

## 6. Patents

Recombinant rAj-HRP from *Apostichopus japonicus* and its application in the preparation of anti-tumor drugs is filed under patent number ZL202210627179.8.

## Figures and Tables

**Table 1:** The effect of rAj-HRP on the tumor volume of HCT116 xenograft mice (*n* = 5).

Categorization	Tumor Volume (mm^3^)	Inhibition Rate (%)
Model	2189.21 ± 815.67	-
5-Fu (10 mg/kg)	1525.30 ± 917.93	30.33
rAj-HRP (50 μg/kg)	1649.61 ± 665.92	24.65
rAj-HRP (100 μg/kg)	1261.07 ± 565.51	42.40
rAj-HRP (200 μg/kg)	910.51 ± 325.84	58.41

**Table 2:** The effect of rAj-HRP on the tumor weight of HCT116 xenograft mice (*n* = 5).

Categorization	Tumor Weight (g)	Inhibition Rate (%)
Model	2.09 ± 0.42	-
5-Fu (10 mg/kg)	1.38 ± 0.11	34.26
rAj-HRP (50 μg/kg)	1.36 ± 0.38	34.96
rAj-HRP (100 μg/kg)	0.64 ± 0.33	69.63
rAj-HRP (200 μg/kg)	0.49 ± 0.14	76.41

## Data Availability

The original contributions presented in this study are included in the article. Further inquiries can be directed to the corresponding authors.
